# A Retrospective Observational Study Examining Physical Activity Among Adults in the United States With Angina or Coronary Heart Disease

**DOI:** 10.7759/cureus.71574

**Published:** 2024-10-15

**Authors:** Kalyan K Annapureddy, Priyanka Mohnani, Jooyoung Moon, Promit Bachhar, Karine Vartanian, Nabeela Fatima

**Affiliations:** 1 Internal Medicine, Dr. Nandamuri Taraka Rama Rao (NTR) University of Health Sciences, Vijayawada, IND; 2 Cardiology, Government Medical College, Surat, Surat, IND; 3 Internal Medicine, Yonsei Good Doctor Clinic, Seoul, KOR; 4 Internal Medicine, The West Bengal University of Health Sciences, Kolkata, IND; 5 Cardiology, Southern California Hospital Heart Institute, Culver City, USA; 6 Cardiology, Shadan Institute of Medical Sciences, Hyderabad, IND

**Keywords:** behavioral risk factor surveillance system (brfss) database, coronary heart disease, exercise, physical activity, u.s. population

## Abstract

Introduction: Coronary heart disease (CHD) involves inadequate blood supply to the myocardium by the coronary arteries due to the formation of atherosclerotic plaque in the vessel lumen. It has a complex etiopathogenesis. Physical activity (PA) and exercise lead to vascular remodeling and improved endothelial function, which, in turn, improves the arterial blood supply to the myocardium.

Objectives: The study aims to determine the prevalence of self-reported PA among CHD patients in the United States based on demographic, socioeconomic, and healthcare access variables for the year 2021.

Methodology: The data for the study on CHD were extracted using the Behavioral Risk Factor Surveillance System (BRFSS) Web-Enabled Analysis Tool database of the U.S. population. The control variables used broadly include demographics, socioeconomic, and healthcare access.

Results: In 2021, 433,615 people in the USA participated in the BRFSS study. Among them, 22,819 self-identified as having angina or CHD. In the past month, 62.2% of participants with the disease were involved in PA, and 37.8% were not. Among participants without angina or CHD, 76.5% were involved, and 23.5% were not involved in PA in the past month.

Conclusion: This study highlights the need for specific interventions to overcome obstacles preventing PA among CHD patients.

## Introduction

Coronary heart disease (CHD) is a condition in which there is an inadequate supply of blood by the coronary arteries to the heart muscles. It occurs due to occlusion of the coronary arteries by the formation of atherosclerotic plaque in the vessel lumen, resulting in a supply-demand mismatch of oxygen [1]. The most prevalent kind of heart disease is CHD. In 2022, it claimed 371,506 lives [2]. CHD affects about one in 20 persons over the age of 20 [3]. Approximately one in five fatalities in 2022 was attributable to cardiovascular diseases in persons under the age of 65 [2].

Chronic heart disease (CHD) is an inflammatory chronic illness marked by reduced oxygen supply to the heart due to the narrowing of the coronary arteries. Its multifactorial nature and intricate etiopathogenesis are linked to genetic and environmental variables, including physical activity (PA), food, and smoking. Atherosclerosis is the main pathogenic process that causes CHD. The build-up of lipids, cholesterol, fibrous components, and inflammatory chemicals in the walls of the major arteries is the hallmark of this quiet, chronic, and progressive process [4]. It has been demonstrated that PA lowers the overall incidence of CHD. To protect the heart and blood vessels, it can enhance insulin sensitivity, reduce plasma dyslipidemia, lower blood viscosity, increase endothelial nitric oxide production, and enhance leptin sensitivity. To prevent CHD, the American Heart Association suggests 30 minutes of moderate-intensity exercise five times a week for adults [5]. PA has a significant dose-response relationship to the risk of developing congestive heart failure (CHD), with a 20% risk reduction in men and women who expend ≈1,100 kcal/week. People who participate in leisure-time PA of less than 550 kcal per week nonetheless have a considerably lower risk of CHD [6]. Vascular remodeling occurs in reaction to exercise and PA. This remodeling includes an increase in the capillaries and conduit and resistance arteries' and arterioles' sizes, all of which improve the myocardium's arterial blood supply. Additionally, exercise enhances endothelial function and slows the advancement of coronary stenosis, partly because it has antiatherosclerotic effects on leukocytes and platelets. PA lowers mortality and cardiovascular event rates in CHD patients, as demonstrated by compelling interventional studies [6].

The Behavioral Risk Factor Surveillance System (BRFSS) is a health-related telephone survey conducted by the Centers for Disease Control and Prevention. Data about self-reported PA levels among patients with CHD were taken from the BRFSS database from 2021.

## Materials and methods

BRFSS database

This retrospective original research study was conducted using the BRFSS (full form) database [7]. The BRFSS is a cross-sectional telephone interview survey conducted monthly in all 50 U.S. states. The survey collects health prevalence data using standardized questionnaires for adult U.S. residents. Data were extracted on July 30, 2024. This is nonhuman participant research, as BRFSS contains deidentified, public data. Thus, no ethics committee approval is needed.

Study population and variables

The data were extracted using the BRFSS Web-Enabled Analysis Tool (WEAT) [8]. Cross tabulation for the single year 2021 was selected, and all locations available (50 states, the District of Columbia, and three U.S. territories) were included. For the row variable, the topic "Chronic Health Conditions" was selected, and among the options, "Ever told you had angina or coronary heart disease (CVDCRHD4)" was selected. For the column variable, the topic "Exercise" was selected. Among the options, "During the past month, any physical activities or exercises such as running, calisthenics, golf, gardening, or walking for exercise (EXERANY2)" was selected. Then, control variables were selected one by one, and each cross-tabulation analysis was subsequently processed.

Control variables were broadly classified into demographic, socioeconomic, and healthcare access. The demographic parameters selected were as follows: "Calculated variable for 4-level imputed age category (_AGE_G, 18-24, 25-44, 45-64, 65+)," "Gender (SEX1)," and "Calculated variable for 4-level race (_RACEGR3)." Age was categorized into four groups: "18-24 years," "25-44 years," "45-64 years," and "65 years or older." Gender was categorized into either male or female. Race was categorized into "non-Hispanic White," "non-Hispanic Black," "Hispanic," and "other."

Socioeconomic parameters included "Education level (EDUCA)," "Employment status (EMPLOY1)," and "Annual household income (INCOME3)." Education level was simplified into two categories: "basic" (less than high school education or high school graduate) and "advanced" (attended or graduated college). Employment status was simplified into two categories: "employed" (employed for wages or self-employed) and "not employed" (out of work or homemaker).

The healthcare access parameter included "How long has it been since the last routine check-up (CHECKUP1)." This was simplified into two categories: "within the past one year" and "more than a year ago or never."

Statistical analysis

Descriptive data in numbers and percentages were generated for each variable using cross-tabulation in the WEAT in the BRFFS. The data were stored in Microsoft Excel (Microsoft Corporation, Redmond, WA), and statistical analysis was performed using R version 4.3.1 (R Core Team, Vienna, Austria). The chi-square test assessed statistically significant differences between self-reported angina or CHD prevalence by PA levels. Fisher's exact test was used to assess statistically significant differences between the prevalence of self-reported angina or CHD among age groups, gender, race, education, employment status, and annual income. A p value of <0.05 was considered statistically significant.

## Results

In 2021 and across the United States, 433,615 people participated in the BRFSS study. Out of this, 22,819 (5.3%) self-identified OR answered "Yes" to the question "Ever told you had angina or coronary heart disease (CVDCRHD4)?" These were thus considered to have angina or CHD.

Table 1 shows the prevalence of PA in the study participants. In the past month, 14,192 (62.2%) participants with the disease were involved in PA, whereas 8,627 (37.8%) were not. Among participants who did not have angina or CHD, 314,119 (76.5%) were involved, and 96,677 (23.5%) were not involved in PA in the past month. The chi-square test suggested that PA was significantly associated with a lower prevalence of self-reported angina or CHD in the past month.

**Table 1:** Prevalence of self-reported angina or CHD by PA level Values are mentioned as N (%) ^*^p value of <0.05 is significant CHD: coronary heart disease; PA: physical activity

Parameters	During the past month, any physical activities or exercises such as running, calisthenics, golf, gardening, or walking for exercise (EXERANY2)			p value
Ever told you had angina or CHD	Yes/no	Yes	No	
	Yes	14,192 (62.2%)	8,627 (37.8%)	<0.001^*^
	No	314,119 (76.5%)	96,677 (23.5%)	

Table 2 shows the prevalence of PA levels based on the demographic characteristics of study participants. The level of PA among patients of angina or CHD was highest in the age group 18-24 years (82.9%), male gender (67.8%), and White, non-Hispanic race (63.3%). Based on demographic variables, PA was significantly associated with a lower prevalence of angina or CHD for age groups 25-44, 45-64, and 65+ years, genders male and female, and races White, non-Hispanic, Black non-Hispanic, Hispanic, and others.

**Table 2:** Prevalence of self-reported angina or CHD by PA level based on demographic characteristics of study participants Values are mentioned as N (%) ^*^p value of <0.05 is significant CHD: coronary heart disease; PA: physical activity

Variables	Angina or CHD	N	Physically active	Physically not active	p value (Fisher's exact test)
Age groups					
18-24 years	Yes	82	68 (82.9%)	14 (17.1%)	0.881
	No	25,840	21,610 (83.6%)	4,230 (16.4%)	
25-44 years	Yes	754	528 (70%)	226 (30%)	<0.001^*^
	No	104,955	85,611 (81.6%)	19,344 (18.4%)	
45-64 years	Yes	5,868	3,601 (61.4%)	2,267 (38.6%)	<0.001^*^
	No	143,571	109,552 (76.3%)	34,019 (23.7%)	
65+ years	Yes	16,115	9,995 (62%)	6,120 (38%)	<0.001^*^
	No	136,430	97,346 (71.4%)	39,084 (28.6%)	
Gender					
Male	Yes	13,227	8,963 (67.8%)	4,264 (32.2%)	<0.001^*^
	No	187,909	148,781 (79.2%)	39,128 (20.8%)	
Female	Yes	9,592	5,229 (54.5%)	4,363 (45.5%)	<0.001^*^
	No	222,887	165,338 (74.2%)	57,549 (25.8%)	
Race					
White, non-Hispanic	Yes	18,412	11,648 (63.3%)	6,764 (36.7%)	<0.001^*^
	No	300,778	234,297 (77.9%)	66,481 (22.1%)	
Black, non-Hispanic	Yes	1,422	777 (54.6%)	645 (45.4%)	<0.001^*^
	No	30,968	21,854 (70.6%)	9,114 (29.4%)	
Hispanic	Yes	1,027	581 (56.6%)	446 (43.4%)	<0.001^*^
	No	37,066	25,616 (69.1%)	11,450 (30.9%)	
Other	Yes	1,359	830 (61.1%)	529 (38.9%)	<0.001^*^
	No	32,035	24,722 (77.2%)	7,313 (22.8%)	

Table 3 shows the prevalence of PA based on the socioeconomic characteristics of study participants. The prevalence of PA among patients with angina or CHD was highest in participants with advanced education level (68.1%), employed (74%), and high income >150,00$ (84.4%). Based on socioeconomic characteristics, PA was significantly associated with a lower prevalence of angina or CHD for basic education and advanced education levels, employed and not employed status, and low-income, mid-income, and high-income categories.

**Table 3:** Prevalence of self-reported angina or CHD by PA level based on socioeconomic characteristics of study participants Values are mentioned as N (%) ^*^p value of <0.05 is significant Basic education: never attended school or only kindergarten, grades 1-8 (elementary), grades 9-11 (some high school), grade 12, or GED (high school graduate) Advanced education: college one year to three years (some college or technical) and college four years or more (college graduate) Employed: employed for wages and self-employed Not employed: out of work for one year or more, out of work for less than one year, a homemaker, and so on CHD: coronary heart disease; PA: physical activity; GED: general education development

Angina or CHD	N	Physically active	Physically not active	p value (Fisher's exact test)	
Education level					
Basic education	Yes	8,425	4,392 (52.1%)	4,033 (47.9%)	<0.001^*^
	No	126,790	82,178 (64.8%)	44,612 (35.2%)	
Advanced education	Yes	14,303	9,747 (68.1%)	4,556 (31.9%)	<0.001^*^
	No	281,837	230,370 (81.7%)	51,467 (18.3%)	
Employment status					
Employed	Yes	4,649	3,440 (74%)	1,209 (26%)	<0.001^*^
	No	218,067	177,361 (81.3%)	40,706 (18.7%)	
Not employed	Yes	17,874	10,570 (59.1%)	7,304 (40.9%)	<0.001^*^
	No	185,092	131,097 (70.8%)	53,995 (29.2%)	
Annual income					
Low income	Yes	10,511	5,726 (54.5%)	4,785 (45.5%)	<0.001^*^
	No	137,985	92,703 (67.2%)	45,282 (32.8%)	
Mid income: from $50,000 to $150,000	Yes	6,538	4,770 (73%)	1,768 (27%)	<0.001^*^
	No	147,473	122,506 (83.1%)	24,967 (16.9%)	
High income: >$150,000	Yes	1,058	893 (84.4%)	165 (15.6%)	<0.001^*^
	No	37,561	34,213 (91.1%)	3,348 (8.9%)	

Table 4 shows the prevalence of PA based on time since the last routine check-up of study participants. Among participants with routine check-ups within the past one year, 12,825 (62%) participants with angina or CHD were physically active compared to 312,469 (75.8%) participants without angina or CHD. This difference was statistically significant. Among patients with routine check-ups of more than one year, 1,229 (65.7%) participants with angina or CHD were physically active compared to 73,531 (79.1%) participants without angina or CHD. This difference was statistically significant. Irrespective of the time since the last routine check-up (within the last one year or more than a year ago), PA in the last one month was significantly associated with a lower prevalence of angina or CHD.

**Table 4:** Prevalence of self-reported angina or CHD by PA level based on time since last routine check-up Values are mentioned as N (%) ^*^p value of <0.05 is significant Within the past one year: within the past year (1-12 months ago) More than a year ago or never: within the past two years (1-2 years ago), within the past five years (2-5 years ago), five or more years ago, and never CHD: coronary heart disease; PA: physical activity

Angina or CHD based on time since the last routine check-up	Yes/no	Total (N)	Physically active, N (%)	Physically not active, N (%)	p value (Fisher’s exact test)
Within the past one year	Yes	20,699	12,825 (62%)	7,874 (38%)	<0.001^*^
	No	312,469	236,929 (75.8%)	75,540 (24.2%)	
More than a year ago or never	Yes	1,871	1,229 (65.7%)	642 (34.3%)	<0.001^*^
	No	92,952	73,531 (79.1%)	19,421 (20.9%)	

## Discussion

PA plays the most crucial role in CHD. It came to light as a major risk factor through the first empirical investigation undertaken by Morris et al. [9], who stated PA reduces the occurrence of CHD. PA comes with a wide variety of health benefits to children and adults, and evidence indicates that the more the PA, the more the benefits [10]. It is well known that PA improves lipid profiles, body weight, insulin sensitivity, and blood pressure, all of which are protective factors. Additionally, it might enhance coronary blood flow and endothelial function. Positive changes in inflammatory and hemostatic variables might be linked to it [11,12]. Lack of regular PA is associated with an increased risk of premature mortality from CHD [13]. Likewise, PA impacts CHD in various dimensions, from being a risk factor to determining its prognosis.

In this view, the authors conducted an observational retrospective study on June 20, 2024, using the BRFSS database to statistically analyze and assess self-reported PA levels among patients with CHD in the United States in 2021.

Among 433,615 surveyed, 5.2% (22,819) were reported to have CHD. Among those with CHD, 62.2% (14,192) were involved in PA. However, 75.7% (314,119) of people without CHD are involved in PA. The possible reason for the findings of comparatively reduced PA among people with CHD has been explained in a study, which showed patients with CHD are sicker, more comorbid, and more anxious about new coronary events and thus not fit to exercise [14]. The findings of increased PA among non-CHD people may be attributed to the fact that regular PA itself could have reduced their risk of developing CHD, considering that they have been physically active from an earlier age before developing CHD, as suggested by Booth et al. [15].

In the surveyed population with CHD, when demographics were considered, PA was lowest among the females 53.61% (5,229), the older age group that is more than 65 years of age 62% (9,995), and black and non-Hispanic 54.6% (777). Similarly, few recent studies have shown that woman has a poorer level of PA than men [16,17]. Several studies done previously around the world have also reported PA differences between genders. These differences have been attributed to the low participation of girls in organized sports, such as body weight, fitness, and boy's preferences for higher intensity activities. In addition, socioeconomic factors at the individual level and gender roles have been identified as contributing factors [18]. Apart from reproductive aging, the hormonal and metabolic change characteristic of the menopausal transition contribute to poorer performance-based physical activity, especially in postmenopausal women [19].

The National Health Interview Survey suggests that limitations in activities of daily living increase the burden for midlife adults [20]. This is mainly attributed to changes in bodily composition and inflammatory changes, social demographic factors, and a decline in mental and physical health due to aging [21].

Women have a longer life expectancy than men [22]. Although CHD is more common in midlife, hypothetically, women still have more years to live. Physical inactivity is also more common among females, which is also consistent with our study. Therefore, from midlife, women may live longer years with disability. Therefore, it is important to encourage PA among women earlier [21].

In our study, the level of PA among the patients with CHD was highest among people with advanced education 68.1% (9,747), employed 74% (3,440), and high-income people 84.5% (893).

A recent study done in 2021 showed that education level positively affects self-reported health level and PA. As people with higher education levels are more economically stable, their subjective health levels can be positively influenced by engaging in affordable PAs [23].

Of people with CHD who had their last routine check-up within the past year, 62% (12,825) are PA. People who had done their last routine check-up more than one year ago with CHD and PA were 65.7% (12,825).

Strength, limitation, and scope for future study

While this study is important and has significant strengths, it also has several limitations. Since this was a telephone survey, individuals without access to landlines, those not using landlines, or those not at home were not contacted. The survey was conducted in English and a few other languages, meaning that individuals who did not speak these languages, potentially belonging to underrepresented groups, were excluded from the study.

The data collected were self-reported, rather than based on actual medical history taken at clinics or through examinations, which introduces the risk of recall bias. The BRFSS dataset does not provide details on the type or severity of CHD, how many months prior the participants developed the disease, or any consequences of the disease. These factors could influence the PA status of the participants.

Additionally, the survey only inquired about PA levels in the past month. It is uncertain whether a one-month period of PA can cause significant improvement in CHD prognosis. The survey also could not establish when the onset of CHD occurred or when participants became physically active or inactive. Being a retrospective observational study, this research can only establish associations, not causations.

## Conclusions

This study highlights the need for targeted interventions to address the barriers to PA among patients with CHD. Increasing PA has been shown in several studies to improve cardiovascular outcomes and quality of life in CHD patients. Ongoing research, such as the European Prospective Investigation into Cancer and Nutrition study and other cohort studies, have reported positive associations between regular PA and reduced CHD progression.

Future research should focus on prospective, large-scale studies to further evaluate how specific types and intensities of PA can modify the disease course in CHD patients. Such studies can inform clinical guidelines to optimize PA levels and ultimately improve patient outcomes. By aligning with current research and addressing these gaps, we can better support individuals with CHD in achieving long-term health benefits.
